# Heterologous production of raspberry ketone in the wine yeast *Saccharomyces cerevisiae* via pathway engineering and synthetic enzyme fusion

**DOI:** 10.1186/s12934-016-0446-2

**Published:** 2016-03-04

**Authors:** Danna Lee, Natoiya D. R. Lloyd, Isak S. Pretorius, Anthony R. Borneman

**Affiliations:** The Australian Wine Research Institute, PO Box 197, Adelaide, SA 5064 Australia; Macquarie University, Sydney, NSW 2109 Australia; Department of Genetics and Evolution, University of Adelaide, Adelaide, SA 5000 Australia

**Keywords:** Phenylpropanoid, Raspberry ketone, Synthetic engineering, Wine yeast

## Abstract

**Background:**

Raspberry ketone is the primary aroma compound found in raspberries and naturally derived raspberry ketone is a valuable flavoring agent. The economic incentives for the production of raspberry ketone, combined with the very poor yields from plant tissue, therefore make this compound an excellent target for heterologous production in synthetically engineered microbial strains.

**Methods:**

A de novo pathway for the production of raspberry ketone was assembled using four heterologous genes, encoding phenylalanine/tyrosine ammonia lyase, cinnamate-4-hydroxlase, coumarate-CoA ligase and benzalacetone synthase, in an industrial strain of *Saccharomycescerevisiae*. Synthetic protein fusions were also explored as a means of increasing yields of the final product.

**Results:**

The highest raspberry ketone concentration achieved in minimal media exceeded 7.5 mg/L when strains were fed with 3 mM p-coumaric acid; or 2.8 mg/L for complete de novo synthesis, both of which utilized a coumarate-CoA ligase, benzalacetone synthase synthetic fusion protein that increased yields over fivefold compared to the native enzymes. In addition, this strain was shown to be able to produce significant amounts of raspberry ketone in wine, with a raspberry ketone titer of 3.5 mg/L achieved after aerobic fermentation of Chardonnay juice or 0.68 mg/L under anaerobic winemaking conditions.

**Conclusions:**

We have shown that it is possible to produce sensorially-relevant quantities of raspberry ketone in an industrial heterologous host. This paves the way for further pathway optimization to provide an economical alternative to raspberry ketone derived from plant sources.

**Electronic supplementary material:**

The online version of this article (doi:10.1186/s12934-016-0446-2) contains supplementary material, which is available to authorized users.

## Background

Plant natural compounds represent a large, chemically-diverse collection of secondary metabolites, however this diversity is generated from a limited number of conserved pathways (reviewed in Marienhagen et al. [1]). One such class of plant compounds are the phenylpropanoids, which like flavonoids, stilbenes and lignans, are formed from the common metabolic precursor p-coumaric acid via the amino acids phenylalanine and tyrosine.

Raspberry ketone [4-(4-hydroxyphenyl)butan-2-one] is a phenylpropanoid that is found in many fruits, berries and vegetables, including raspberries, blackberries, grapes and rhubarb. As the name suggests, it is considered a major impact sensory molecule in raspberry, along with other volatile aroma compounds such as monoterpenes, β-damascenone and α- and β-ionone [2, 3]. Naturally-derived raspberry ketone is a valuable flavoring agent (US$3000/kg), as plant-derived yields of this compound are very low, even from raspberries (1–4 mg/kg) [3, 4]. Raspberry ketone can also be derived through chemical means, however this synthetic compound attracts a far lower price (US$58/kg) than the naturally-derived form [4].

The economic incentives for the production of raspberry ketone, combined with the very poor yields from plant tissue, therefore make this compound an excellent target for production via the use of synthetically engineered microbial strains. Previous work has shown that it is possible to produce raspberry ketone from p-coumaric acid in heterologous systems such as *Escherichia coli* (5 mg/L) and *S. cerevisiae* (trace amounts) [5]. However, de novo production of raspberry ketone, without the need for precursor addition, has not yet been demonstrated.

In order to establish a heterologous system for raspberry ketone production, a de novo biosynthetic route, comprising four separate enzymatic activities, has been engineered into *S. cerevisiae*. Furthermore in order to optimize the production of this valuable aroma compound, synthetic protein fusions were explored and found to increase final concentrations of raspberry ketone over fivefold. Finally, as the metabolic engineering was performed in a wine strain of *S. cerevisiae*, we show that this engineered strain is capable of synthesizing raspberry ketone at concentrations almost two orders of magnitude above its predicted sensory threshold in Chardonnay grape juice under standard winemaking conditions, while retaining the ability to complete fermentation.

## Results and discussion

### Biosynthesis of raspberry ketone from p-coumaric acid

**Fig. 1:**
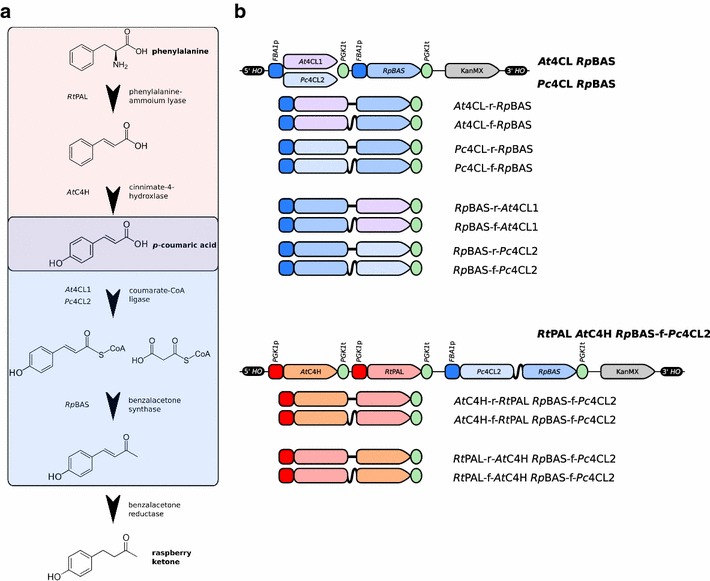
Engineering the raspberry ketone biosynthetic pathway in *S. cerevisiae*. **a** The phenylpropanoid pathway begins with the conversion of phenylalanine to p-coumaric acid via cinnamate or directly from tyrosine to p-coumaric acid (*pink box*). Conversion of p-coumaric acid to raspberry ketone requires three additional enzymatic steps including a condensation reaction between coumaroyl-CoA and malonyl-CoA. Heterologous production of raspberry ketone can be accomplished by the final three enzymatic reactions, if microbial cells are supplied with exogenous p-coumaric acid (*blue box*). The heterologous enzymes used for each reaction in this study are also listed. **b** Expression constructs used in this study for the production of raspberry ketone. Rigid and flexible linker sequences that were used for the protein fusions are represented by *bold black lines* (straight and wavy, respectively)

The production of raspberry ketone from p-coumaric acid requires the action of two heterologous enzyme activities, a coumarate-CoA ligase (4CL) and benzalacetone synthase (BAS), as yeast has been shown to natively display efficient benzalacetone reductase (BAR) activity [5] (Fig. 1a).

Based upon previous literature, two different 4CL genes were chosen for investigation, *A.thaliana* (*At*4CL1, GenBank: AAA82888.1) and parsley (*Petroselinumcrispum*; *Pc*4CL2, GenBank: CAA31697.1) [6, 7]. For BAS activity, the rhubarb (*Rheumpalmatum*) BAS (*Rp*BAS, GenBank: AAK82824.1) carrying a S338 V mutation was selected, as this enzyme provides high BAS activity without the co-production of naringenin [8, 9].

**Fig. 2:**
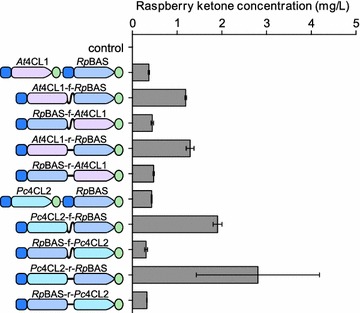
Biosynthesis of raspberry ketone from p-coumaric acid during anaerobic fermentation. Codon optimized genes encoding coumarate CoA ligase (4CL) from either *A. thaliana* (*At*4CL1, *pink*) or *P. crispum* (*Pc*4CL2, *blue*) and benzalacetone synthase (BAS) from *R.* *palmatum* (*Rp*BAS) were integrated at the *HO* locus of *S. cerevisiae* as either two independent genes or as a single ORF fused by either a flexible (f) or rigid (r) amino acid linker. Levels of raspberry ketone were assessed following 5 days growth at 22 °C in air-lock flasks in synthetic grape juice medium, supplemented with 3 mM p-coumaric acid and assessed for raspberry ketone production via LC/MS

Codon optimized versions of the heterologous genes were synthesized and the two different 4CL and BAS pairs (*At*4CL1 *Rp*BAS and *Pc*4CL2 *Rp*BAS) were integrated in the *HO* locus of AWRI2975 (*TDH3*p:*ACC1*) in a tandem arrangement, with each gene driven from a separate copy of the highly-fermentation expressed promoter of *FBA1* [10, 11] (Fig. 1b). The *HO* locus was chosen as integration at this genomic location has been shown to be phenotypically neutral [12, 13]. Fermentations, supplemented with p-coumaric acid (3 mM final concentration), were performed with these two strains, with raspberry ketone levels of 0.37 ± 0.01 mg/L and 0.43 ± 0.01 mg/L observed for the *At*4CL1 *Rp*BAS and *Pc*4CL2 *Rp*BAS constructs, respectively (Fig. 2). While relatively low, these levels are nevertheless >30–300 fold above established sensory thresholds for raspberry ketone in aqueous solutions (0.001–0.01 mg/L) [2, 14].

In order to attempt to increase the levels of raspberry ketone produced, a synthetic fusion enzyme strategy was employed, as this has been shown to increase yields of other heterologous metabolites, presumably through substrate funneling [15, 16]. Short protein linkers that are predicted to have either a flexible (VDGGSGR) or rigid (VDEAAAKSGR) conformation [16] were used to fuse the coding regions of the 4CL and BAS enzymes in both tandem orientations (4CL-*Rp*BAS and *Rp*BAS-4CL), using either the *A. thaliana* or *P. crispum* 4CL genes (Fig. 1b). The production of raspberry ketone was then assessed for these four different constructs compared to the binary gene systems (Fig. 2). While the type of linker (flexible or rigid) did not significantly affect the observed levels of raspberry ketone, fusing the two genes in the 4CL-*Rp*BAS orientation provided 3.5- and 6.5-fold increases in the levels of raspberry ketone produced with the *A. thaliana* (1.29 ± 0.09 mg/L) or *P. crispum* (2.81 ± 1.38 mg/L) 4CL genes, respectively. However this effect was specific to the orientation of the fusion protein, as the *Rp*BAS-4CL fusions provided no significant difference in raspberry ketone levels compared to the two independent genes.

### Effect of oxygen on the production of raspberry ketone

**Fig. 3:**
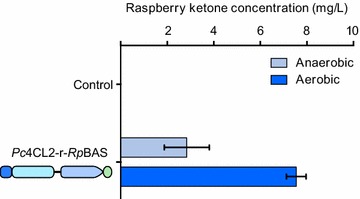
The Effect of oxygen on the production of raspberry ketone. Strains containing the *P. crispum* coumarate CoA ligase 2 (*Pc*4CL2) and benzalacetone synthase (BAS) from *R.* *palmatum* (*Rp*BAS) ORFs fused by rigid (r) amino acid linker were fermented in either airlock flasks (anaerobic) or standard flasks (aerobic) in synthetic grape juice medium, supplemented with 3 mM p-coumaric acid and assessed for raspberry ketone production via LC/MS

As the *Pc*4CL2-*Rp*BAS fusion constructs were shown to have the highest activity in anaerobic fermentation, the *Pc*4CL2-r-*Rp*BAS strain was evaluated for raspberry ketone production under aerobic growth conditions, so that the effect of oxygen could be assessed (Fig. 3). The aerobic fermentation resulted in a significant increase in raspberry ketone production of 2.6-fold (7.54 ± 0.42 mg/L).

### De novo biosynthesis of raspberry ketone

While the aforementioned strains are capable of producing significant levels of raspberry ketone, they require the exogenous addition of the pathway precursor p-coumaric acid. In order to engineer the de novo production p-coumaric acid in *S. cerevisiae* from the aromatic amino acid precursors phenylalanine and tyrosine, three enzyme activities were required (Fig. 1), phenylalanine ammonia lyase (PAL), tyrosine ammonia lyase (TAL) and cinnamate-4-hydroxylase (C4H), with some enzymes, such as PAL from *Rhodosporidiumtoruloides* providing both PAL and TAL activities [17]. The *Rhodosporidiumtoruloides* PAL (*Rt*PAL, GenBank: CAD23831.1) and *Arabidopsisthaliana* C4H (*At*C4H, GenBank: AEC08397.1) were therefore chosen as these two enzymes had been shown to function in *S. cerevisiae* to produce p-coumaric acid previously [17, 18].

**Fig. 4:**
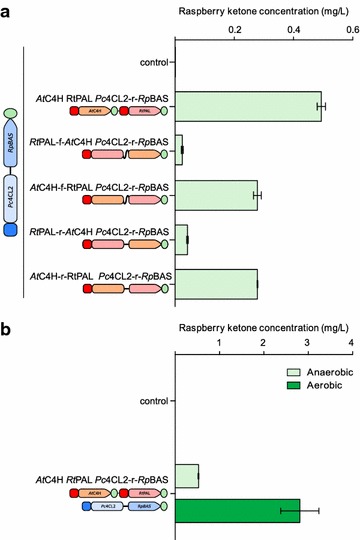
Full de novo biosynthesis of raspberry ketone. **a** Codon optimized genes encoding phenylalanine ammonia lyase from *Rhodosporidium* *toruloides* PAL (*Rt*PAL) and cinnamate-4-hydroxylase from *Arabidopsis* *thaliana* (*At*C4H) were integrated at the *HO* locus of *S. cerevisiae* as either two independent genes or as a single ORF fused by either a flexible (f) or rigid (r) amino acid linker. All strains also contained the *P. crispum* coumarate CoA ligase 2 and benzalacetone synthase from *R.* *palmatum* ORFs fused by a flexible linker (*Pc*4CL2-f-*Rp*BAS), positioned adjacently in the *HO* locus. Levels of raspberry ketone were assessed following five days growth at 22 °C in air-lock flasks in synthetic grape juice medium and assessed for raspberry ketone production via LC/MS. **b** A strain containing the *Rhodosporidium* *toruloides* PAL (*Rt*PAL) and cinnamate-4-hydroxylase from *Arabidopsis* *thaliana* (*At*C4H) as separate ORFs in addition to the *P. crispum* coumarate CoA ligase 2 and benzalacetone synthase from *R.* *palmatum* ORFs fused by rigid linker (*Pc*4CL2-r-*Rp*BAS) were fermented in either airlock flasks (anaerobic) or standard flasks (aerobic) in synthetic grape juice medium and assessed for raspberry ketone production via LC/MS

The *Rt*PAL and *At*C4H enzymes were therefore codon optimized and assembled as a binary gene system under the control of the highly-fermentation expressed *S. cerevisiae PGK1* promoter [11]. These two genes were then integrated into *HO* locus of *S. cerevisiae* in combination with the *Pc*4Cl2-r-*Rp*BAS fusion gene (Fig. 1b). In anaerobic ferments, the binary gene pair produced 0.49 ± 0.01 mg/L of raspberry ketone, representing 18 % of the yield of the *Pc*4Cl2-r-*Rp*BAS strain supplemented with p-coumaric acid (2.81 ± 1.38 mg/L) (Fig. 4a). As performed for the *Pc*4CL2-*Rp*BAS system, protein fusions were explored using combinations of *At*C4H, *Rt*PAL and either the flexible or rigid linkers to determine if this could result in higher de novo production levels (Fig. 1b). As seen in the *Pc*4CL2-*Rp*BAS fusions, no significant difference was observed between the use of the rigid and flexible linkers, however unlike the previous system, all four fusions (two linkers × two gene orders) performed significantly worse than the binary gene system (Fig. 4a). Furthermore, the two different fusion orders produced vastly different results, as while the *At*C4H-*Rt*PAL fusions reduced activity by ~50 % (0.28 ± 0.01 mg/L), the *Rt*PAL-*At*C4H fusions almost totally abolished enzyme activity (0.02–0.04 mg/L).

Having established the de novo production of raspberry ketone under anaerobic conditions, the effect of oxygen was also determined (Fig. 4b). As also observed in the precursor-fed fermentations, aerobic growth resulted in a significant increase in raspberry ketone levels, to 2.81 ± 0.43 mg/L (fivefold).

In order to ensure that the production of raspberry ketone was not impacting cell growth or fermentation ability, the fermentation kinetics of highest raspberry ketone producing strain (*At*C4H *Rt*PAL *Pc*4Cl2-r-*Rp*BAS) were compared to both AWRI2975 and AWRI2975 containing the integrated empty vector control under both aerobic and anaerobic growth (Additional file 1: Fig. S1). While the *At*C4H *Rt*PAL *Pc*4Cl2-r-*Rp*BAS strain displayed a slight delay in total sugar consumption, it was able to finish fermentation efficiently, reaching dryness at the same time point as the parental and control strains.

### De novo biosynthesis of raspberry ketone in chardonnay ferments

**Fig. 5:**
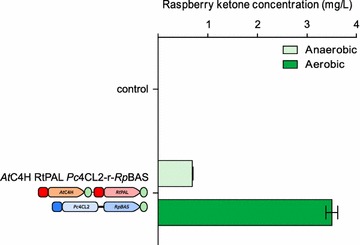
De novo production of raspberry ketone during wine fermentation. A strain containing the *Rhodosporidium* *toruloides* PAL (*Rt*PAL) and cinnamate-4-hydroxylase from *Arabidopsis* *thaliana* (*At*C4H) as separate ORFs in addition to the *P. crispum* coumarate CoA ligase 2 and benzalacetone synthase from *R.* *palmatum* ORFs fused by rigid linker (*Pc*4CL2-r-*Rp*BAS) was fermented in Chardonnay grape juice in either airlock flasks (anaerobic) or standard flasks (aerobic) until dryness and assessed for raspberry ketone production via LC/MS

Given that a wine strain of *S. cerevisiae* was used for this pathway engineering (AWRI1631) [19], the growth and production of raspberry ketone by the *At*C4H *Rt*PAL *Pc*4Cl2-r-*Rp*BAS strain was assessed in Chardonnay grape juice under both aerobic and anaerobic (winemaking) conditions (Fig. 5). There was no significant difference in the levels of raspberry ketone between the synthetic and real grape juice, with 0.68 ± 0.02 and 3.49 ± 0.12 mg/L of raspberry ketone produced under anaerobic and aerobic conditions, respectively.

## Conclusions

Raspberry ketone represents a highly desirable plant-derived compound, providing a major positive aroma in raspberries. The fermentation-derived production of significant amounts of raspberry ketone has been achieved through the introduction of four heterologous genes into *S. cerevisiae*. This represents the first description of de novo production of raspberry ketone by yeast.

Furthermore, by engineering a synthetic fusion protein between 4CL and BAS, it was possible to increase the levels of raspberry ketone approximately sixfold, with the maximum de novo production equating to levels generally observed in fresh raspberries. Synthetic fusion proteins have been used extensively in metabolic engineering, where it is thought to allow for increased flux through substrate channeling [20]. In this system, we observed that while proteins were very sensitive to fusion and sometimes, even the particular orientation of the fusion, they were largely insensitive to the predicted secondary structure of the linker (rigid or flexible). This effect was also observed in [16], where the order of farnesyl diphosphate synthase and patchoulol synthase in a protein fusion was shown to be important, but activity was largely insensitive to changes in linker length or secondary structure.

Aerobic culturing consistently resulted in a significant increase in the final concentration of raspberry ketone produced, regardless of the media. Aerobic fermentation would therefore be favored in situations where the maximum amount of raspberry ketone is desired, such as when it is to be isolated as a purified natural flavor product. Purified compounds, such as those produced through the use of engineered microbial strains, are classified as natural flavor compounds and can therefore attract far higher prices than chemically synthesized compounds [4]. Further optimization of raspberry ketone biosynthesis could make the microbial production of purified raspberry ketone economically viable.

The final levels of raspberry ketone produced under winemaking conditions well exceeded the sensory threshold of this compound (0.001–0.01 mg/L in water) [2, 14], while the strain was still able to efficiently complete fermentation. While this is a genetically-modified strain and is therefore unavailable for use in most winemaking countries, it would nevertheless be capable of producing a sensorily-augmented product if used in wine fermentation. The production of plant-derived compounds such as raspberry ketone or monoterpenes [21, 22], by engineered yeast highlights the potential for synthetic biology and metabolic engineering to impart novel fermentation properties in industrial strains.

## Methods

### ACC1 *promoter modification*

To remove acyl-CoA feedback inhibition on the *ACC1* gene during fatty acid biosynthesis, the strain AWRI2975 was produced by inserting the constitutive promoter of the *S. cerevisiaeTDH3* gene immediately upstream of the *ACC1* start codon in the haploid wine strain AWRI1631 [19] using the delitto perfetto method [23, 24].

### Gene synthesis

*Rhodosporidiumtoruloides* PAL (*Rt*PAL, GenBank: CAD23831.1), *Arabidopsisthaliana* C4H (*At*C4H, GenBank: AEC08397.1) and 4CL1 (*At*4CL1, GenBank: AAA82888.1), *Petroselinumcrispum* 4CL2 (*Pc*4CL2, GenBank: CAA31697.1), and *Rheumpalmatum* BAS (RpBAS, GenBank: AAK82824.1) carrying S338 V mutation were selected to reconstruct the raspberry ketone biosynthesis pathway [9]. Predicted protein sequences of each gene were obtained from GenBank, converted into nucleotide sequences and codon optimized for expression in *S. cerevisiae* (GeneArt). A *Not*I restriction site was added before the start codon of each gene, a *Sal*I site was placed immediately in front of the stop codon, and a *Bam*HI site was added after the stop codon to facilitate cloning and the construction of fusion enzymes.

### Cloning of raspberry ketone pathway and pathway integration

Individual synthetic genes were cloned into pCV2-BB entry plasmid, which is a non-replicating variant of pCV3 [25] using the flanking *Not*I and *Bam*HI sites. Two gene and four gene daisy chains were created by ligating *Eco*RI-*Xba*I digested inserts into *Eco*RI- *Spe*I digested vectors. Fusion genes were created by ligating hybridized flexible (VDGGSGR) or rigid (VDEAAAKSGR) linker oligonucleotides [16] carrying 5′ *Sal*I and 3′ *Not*I overhangs with *Eco*RI-*Sal*I digested pCV2-BB plasmids containing the 5′ genes and *Eco*RI-*Not*I digested plasmids containing the 3’ genes.

The integrating plasmid pCV2-BB-HO1 was created by Gibson assembly [26] through the addition of two 150 bp flanking segments homologous to the *S. cerevisiaeHO* locus with *Xho*I sites incorporated at the termini. Raspberry ketone pathways constructed in pCV2-BB entry plasmids were sub-cloned into pCV2-BB-HO1 plasmids, which were digested with *Xho*I before being used in yeast transformation.

### Culture condition and fermentation

All yeast strains were maintained on YPD agar plates containing 200 mg/L G418. Overnight cultures of the yeast strains were grown in 2 mL YPD containing 200 mg/L G418 at 28 °C. The overnight YPD cultures were inoculated 1/100 into 5 mL 50:50 synthetic grape juice [27]: YPD containing 200 mg/L G418 and were grown at 28 °C for 24 h to an optical density (OD) of 2.60 ± 0.05. The overnight synthetic grape juice-YPD cultures were then used as 1:100 starter cultures for ferments. Anaerobic fermentation of synthetic grape juice (100 mL) were performed in triplicate in 200 mL Erlenmeyer flasks fitted with water-filled airlocks, incubated at 22 °C with shaking at 130 rpm; aerobic fermentations were performed in triplicate in 200 mL Erlenmeyer flasks covered with aluminium foil.

For 4CL-BAS strains, p-coumaric acid was added to synthetic grape juice ferments 24 h post-inoculation to a final concentration of 3 mM. Ferments were allowed to proceed for 5 days with the sugar concentrations analysed by HPLC. Chardonnay ferments were performed similarly to synthetic grape juice experiments, but without G418 and were allowed to proceed for 8 days.

### LC–MS/MS analysis

Following cold settling of the ferments, the supernatant for each sample was diluted 1 in 20 with Milli-Q water and analyzed by LC–MS/MS. Calibrants were prepared in matrix (diluted 1 in 20 with Milli-Q water) at levels 0, 0.5, 1, 2, 5, 10, 25 and 50 μg/L from a stock solution of raspberry ketone standard (Sigma Aldrich).

An Agilent 1290 Infinity UHPLC coupled with the 6490 QQQ LC–MS with iFunnel technology was used for the quantification of raspberry ketone. Data acquisition and processing was performed using Mass Hunter software version B.06.00 (Agilent, USA). Samples (10 μL) were injected onto a Zorbax Eclipse XDB-C18 Rapid Resolution HT 4.6 mm × 50 mm 1.8 μm (Agilent, USA). The column temperature was 25 °C. The HPLC mobile phases were 0.1 % formic acid (v/v) with 10 mM ammonium formate in water (w/v) (solvent A) and methanol (solvent B). An isocratic gradient was used comprising of 50 % solvent A/solvent B with a flow rate of 0.6 mL/min and a runtime of 5 min. Quantitative analysis was performed using multiple reaction monitoring and ESI positive ionization mode. The following transitions were monitored: m/z 107 → 77 (quantifier), 165 → 107 (qualifier) for raspberry ketone. The following source conditions were used: gas temperature 290 °C; gas flow 14 L/min; nebulizer 40 psi; sheath gas temperature 300 °C; and sheath gas flow 11 L/min. Nitrogen was used as the nebulizer and collision cell gas. The linear calibration range (LCR) was from 0.5 to 50 μg/L in matrix-matched standards. The limit of detection (LOD) was 0.5 μg/L and the limit of quantification was 1 μg/L.

## Additional file

10.1186/s12934-016-0446-2 Fermentation kenetics of during raspberry ketone production. AWRI2975, AWRI2975 containing an empty vector integrated at *HO* (control) and *At*C4H *Rt*PAL *Pc*4Cl2-r-*Rp*BAS (RK) were all used to ferment synthetic Chardonnay juice under either anaerobic (A) or aerobic conditions (B). Absorbance (OD_600_) and residual sugar were both recorded at 24 h intervals for 5 days.

## Abbreviations

4CL: coumarate-CoA ligase
BAS: benzalacetone synthase
BAR: benzalacetone reductase
PAL: phenylalanine ammonia lyase
TAL: tyrosine ammonia lyase
C4H: cinnamate-4-hydroxylase
