# Supplementary figures and images for: Heterologous production of raspberry ketone in the wine yeast Saccharomyces cerevisiae via pathway engineering and synthetic enzyme fusion
Source: Microb Cell Fact. 2016 Mar 4;15:49. doi: 10.1186/s12934-016-0446-2 (PMC4779194; doi:10.1186/s12934-016-0446-2)

**A**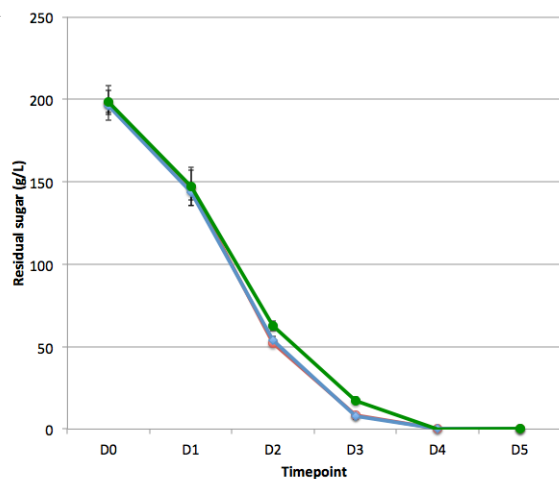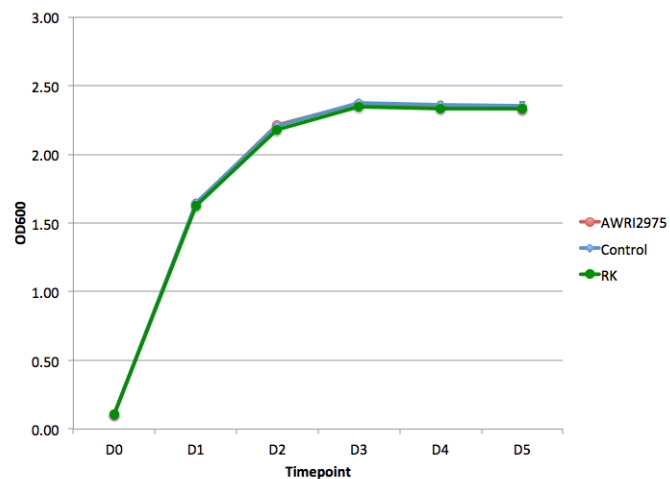**B**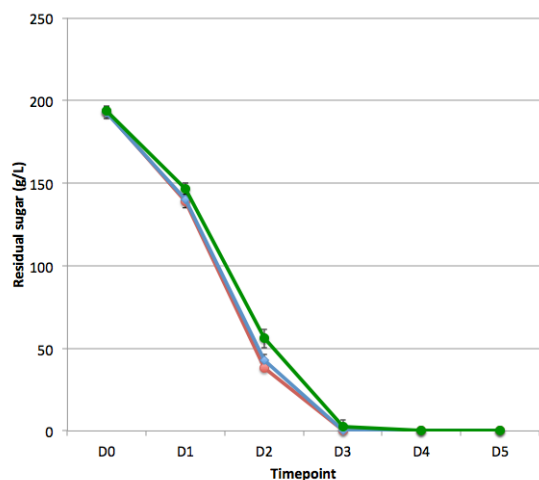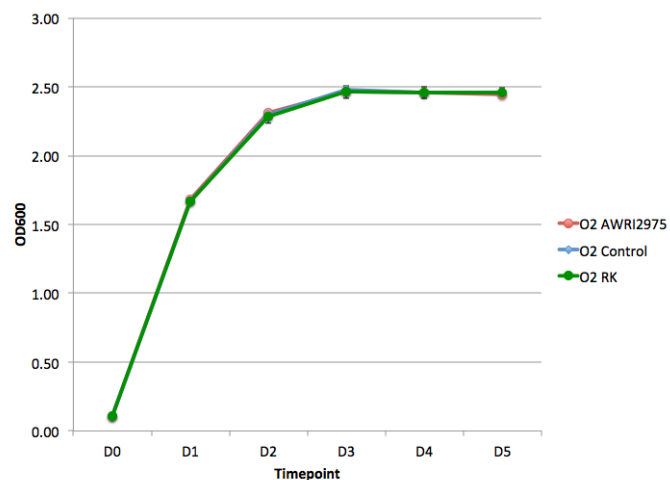

Supplement: Supplementary file 1 — 10.1186/s12934-016-0446-2 Fermentation kenetics of during raspberry ketone production. AWRI2975, AWRI2975 containing an empty vector integrated at HO (control) and AtC4H RtPAL Pc4Cl2-r-RpBAS (RK) were all used to ferment synthetic Chardonnay juice under either anaerobic (A) or aerobic conditions (B). Absorbance (OD600) and residual sugar were both recorded at 24 h intervals for 5 days. [file 12934_2016_446_MOESM1_ESM.pdf]
